# Binary Choice Health State Valuation and Mode of Administration: Head-to-Head Comparison of Online and CAPI

**DOI:** 10.1016/j.jval.2012.09.001

**Published:** 2013-01

**Authors:** Brendan Mulhern, Louise Longworth, John Brazier, Donna Rowen, Nick Bansback, Nancy Devlin, Aki Tsuchiya

**Affiliations:** 1 Health Economics and Decision Science, School of Health and Related Research, University of Sheffield, Sheffield, UK; 2 Health Economics Research Group, Brunel University, London, UK; 3 School of Population and Public Health, University of British Columbia, Vancouver, BC, Canada; 4 Office of Health Economics, London, UK; 5 Department of Economics, University of Sheffield, Sheffield, UK

**Keywords:** CAPI, health state valuation, online

## Abstract

**Background:**

Health state valuation exercises can be conducted online, but the quality of data generated is unclear.

**Objective:**

To investigate whether responses to binary choice health state valuation questions differ by administration mode: online versus face to face.

**Methods:**

Identical surveys including demographic, self-reported health status, and seven types of binary choice valuation questions were administered in online and computer-assisted personal interview (CAPI) settings. Samples were recruited following procedures employed in typical online or CAPI studies. Analysis included descriptive comparisons of the distribution of responses across the binary options and probit regression to explain the propensity to choose one option across modes of administration, controlling for background characteristics.

**Results:**

Overall, 422 (221 online; 201 CAPI) respondents completed a survey. There were no overall age or sex differences. Online respondents were educated to a higher level than were the CAPI sample and general population, and employment status differed. CAPI respondents reported significantly better general health and health/life satisfaction. CAPI took significantly longer to complete. There was no effect of the mode of administration on responses to the valuation questions, and this was replicated when demographic differences were controlled.

**Conclusions:**

The findings suggest that both modes may be equally valid for health state valuation studies using binary choice methods (e.g., discrete choice experiments). There are some differences between the observable characteristics of the samples, and the groups may differ further in terms of unobservable characteristics. When designing health state valuation studies, the advantages and disadvantages of both approaches must be considered.

## Introduction

To conduct health state valuation studies, a range of administration modes can be used. These include face-to-face interviews using paper-and-pencil methods, face-to-face interviews using computer-assisted personal interviews (CAPIs—in which surveys are displayed via a computer interface while an interviewer is present), and online studies. To date, face-to-face interviews using paper-and-pencil methods have been the most widely used mode for collecting health state valuation data and were used to derive preference weights for EuroQol five-dimensional (EQ-5D) questionnaire using the time trade-off (TTO) preference elicitation technique [1] and six-dimensional health state short form (derived from SF-36) using the standard gamble (SG) approach [2,3]. TTO studies employing CAPI methods have also been used to derive EQ-5D questionnaire value sets [4,5]. An online version of the TTO process has been investigated, and some concerns have been found with the approach [6]. TTO and SG tasks in these studies have been “iterative”: TTO or SG of a given health state consists of a series of choices, iterated for different values until a point of indifference is reached for each individual respondent for the state. However, techniques based on binary choice can mean that each choice made by the respondent is independent of the one before. A discrete choice experiment (DCE) is an example of a binary choice technique and has been used in a face-to-face setting [7] and in online settings [8].

Each mode has advantages and disadvantages that may affect the data generated and therefore need to be considered in the design of health state valuation studies. Face-to-face interviews may provide high-quality data with good completion rates and reliability [9] but are expensive and time-consuming to conduct. The use of CAPI means that some of the advantages of online surveys can be exploited in face-to-face environments. For example, CAPIs allow for complex routing of questions, question order randomization, recording of the time taken, and the minimization of errors associated with data entry (which is completed automatically). Online valuation methods have the advantages related to CAPI but are also cheaper to conduct, allow large samples to be achieved in a short space of time, have a flexible sampling frame, and enable a range of background characteristics of nonrespondents to be obtained. The two main disadvantages of online surveys relate to the representativeness of the sample and the quality of the data. People who have access to the Internet may not be representative of the wider population, and answering a survey unsupervised may lead to low-level engagement or poor understanding.

Studies have compared the online and CAPI administration of health state valuation exercises. Comparisons of the online and face-to-face administration of iterative person trade-off (PTO) tasks have found broadly similar results across modes [10,11]. Robinson et al [10], however, found that a greater number of online respondents gave “equivalence” responses, which provided the quickest way to finish each PTO task. Norman et al [6] compared the online and face-to-face (but not CAPI-based) administration of an iterative TTO task and found that the responses differed by administration mode, with those completing the online survey displaying more variation in response. When the results were modeled to generate a value set for the EQ-5D questionnaire, 100 of the 243 health state values were higher in the online group by at least 0.1. Therefore, iterative health state valuation tasks administered online may generate different results from CAPI, but it is not clear whether the difference is due to the mode of administration used or differences in the perception and completion of an iterative task when administered by using different modes of administration.

In addition, it has not been established whether responses to binary choice health state valuation questions are comparable across administration modes. Binary choice questions such as DCE_TTO_, where duration is included as a dimension alongside the health state classification system, can be used to value health states at the aggregate level [8]. Binary choice questions such as DCE with no duration can be used to value health states in “hybrids” with TTO [12,13]. Because conventional health state valuation questions such as SG or TTO based on iteration to identify indifference are effectively made of a series of binary choice questions, a set of independent “snapshot” binary choice questions can be used to value health states at the aggregate level [14]. Comparing the online versus CAPI administration of binary choice questions to investigate the level of equivalence between the modes of administration has direct implications for the better understanding of these methods, and the design of future health state valuation studies using binary choice techniques.

Collecting data online raises concerns about the representativeness of the sample and comparability with data collected by using other methods. Samples recruited online may be biased in terms of unobserved characteristics [15]. There are also concerns about the motive of participation, the level of nonresponse and attrition [16], the reliability and validity of the data generated [17–19], and the level of engagement of respondents. Nevertheless, other studies have found comparability across samples [20,21], in addition to comparability in terms of the reliability and validity of the data generated [22].

This study aimed to compare responses to binary choice versions of health state valuation questions across online and CAPI modes of administration. This was done by administering identical surveys where the only difference was the mode of administration used to collect the data. We hypothesized that the responses to different types of binary choice valuation questions do not differ across modes. To investigate issues around the representativeness of samples recruited for face-to-face and online studies, we also compare the demographic characteristics and self-reported health status of the samples and those of the general population.

## Methods

### Survey

**Fig. 1:**
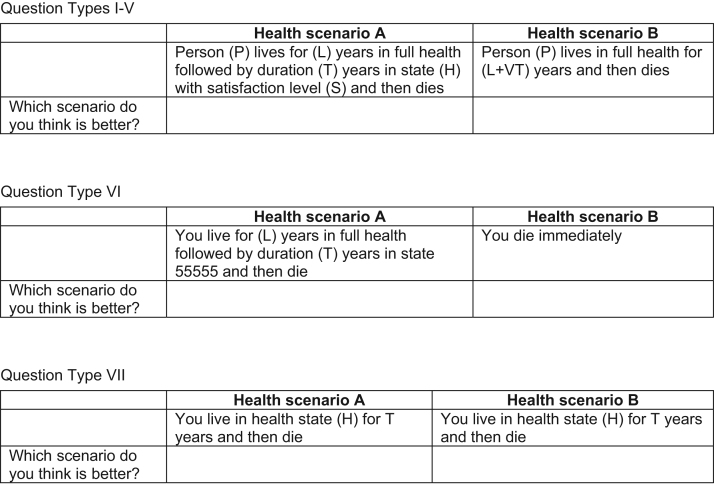
Basic binary choice question format used in the survey.

The online and CAPI survey data sets used in this study contained identical demographic and binary choice questions in the same order. Both data sets were part of a wider research project where binary choice questions were used to investigate methodological issues related to health state valuation. Seven different “types” of binary choice questions were included in the online survey, and the basic format of the questions is described in Figure 1. The questions included a number of experimental attributes that can be varied across hypothetical health states. These include health state experienced (H), time spent in the health state (T), lead time spent in full health before the health state (L), perspective of the person experiencing the health state (P), and the level of satisfaction with health while experiencing the health state (S). The seven question types were designed to examine a number of methodological issues typically involved in health state valuations, namely, that health state preferences are independent of 1) the duration of the state, 2) whose health it is (i.e., perspective), 3) the length of “lead time” (a mechanism to value all states on the same scale including those that are worse than being dead), 4) when health states take place, and 5) the satisfaction associated with the state. Further topics addressed were 6) the lowest health state value that can be valued by using lead time and 7) health state valuation by using DCEs with duration as an attribute (DCE_TTO_). Not all the attributes above vary across each question (see Table 1 for the attribute combinations used across each question type and Tsuchiya and Mulhern [23] for more details).

The online survey had 15 versions, most of which contained only three of the seven types of questions. One version, however, contained all seven question types, and this is used in the study reported here. The CAPI survey matched this particular online version in terms of questions and sample size. In other words, the data used in this comparison study have a wide coverage of question type, but it is not intended that they are used to address any of the methodological issues themselves. In this version, 12 questions were administered across three question modules (the time taken to complete each module was recorded). Module 1 included five type I questions, module 2 included one of the question types II–VI (so five questions in total), and module 3 included two DCE_TTO_ questions (type VII; two subversions of module 3 were included for both the online and the CAPI survey so that four different questions of this type were used overall). The health states used were based on the EQ-5D-5L [24]. The EQ-5D-5L describes health status across five dimensions—mobility, self-care, usual activities, pain/discomfort, and anxiety/depression—each with five response levels—no, slight, moderate, severe, and extreme/unable.

Each survey began by providing study information, and this was followed by a compulsory informed consent page. Respondents were then asked a series of demographic questions and completed self-reported health status (on a 5-point scale, with scores ranging from “excellent” to “poor”), health and life satisfaction questions (on a 10-point scale, with scores ranging from “completely satisfied” to “completely dissatisfied”), and the EQ-5D-5L before completing the 12 binary choice valuation questions.

### Recruitment and the Sample

To achieve a comparison of the two modes of administration as they would happen in the real world, the CAPI and online samples were recruited separately following procedures employed in typical surveys.

For the online survey, respondents were sourced from an existing Internet panel and were selected following set quotas based on the UK general population across five age groups—18 to 24, 25 to 34, 35 to 44, 45 to 54, 55 to 64—and sex. Invitations were sent out via e-mail. Potential respondents were screened out before starting the experimental questions if the relevant quota for age and sex was already complete or after the completion if they completed the survey in less than the minimum imposed time limit of 5 minutes (which was chosen after inspection of the completion times from the initial launch of the survey). The online survey described in this study was 1 of 15 different online surveys that aimed for an overall achieved sample of 3000 (∼200 respondents per version). The questions included in the version described here were identical to those used in the CAPI survey, and therefore a subset of respondents from the overall sample is considered in this study.

For the CAPIs, recruitment followed set quotas for age and sex based on the UK general population and scaled down for an achieved sample of 200. This attempted to ensure the overall comparability of the sample characteristics across the administration modes. Participants were recruited by knocking on 1 in every 10 doors in randomly selected postcodes in five UK areas. The survey was presented to respondents on a laptop, and the interviewer read out all the questions and text on the screen and recorded the response given by the respondent. This was done in a one-to-one setting. The same minimum completion time of 5 minutes was imposed, and participants were able to stop the survey at any time.

### Analysis

Background characteristics, self-reported health status, and time taken to complete the survey were compared across the two samples by using chi-square analysis, Mann-Whitney *U* analysis, and analysis of variance. The background characteristics of the overall, CAPI, and online samples were also compared with the general population of England and Wales by using statistics extracted from the 2001 UK census [25] for 18- to 64-year-olds. Comparisons of the proportion of respondents who chose scenario B by sample and by the seven binary choice question types were carried out, with statistical significance indicated by *P* values less than 0.05.

Comparisons of the proportions of respondents choosing scenario B using a range of completion time cutoff points (i.e., 5, 6, 7, and 8 minutes) were also investigated by using chi-square analysis. We also compared the responses for different groups of respondents defined by the time taken to complete each of the three binary choice question modules. Two groups were defined for each module: group 1 included those completing the module in less than the median time taken to complete the module (and also the overall survey in more than 5 minutes) and group 2 included those completing the module in more than or equal to the median time taken to complete the module (and the overall survey in more than 5 minutes). The median time was used as the cutoff to allow for a reasonably equal split between the groups.

Probit regressions were used to explore the determinants of the propensity to choose scenario B for each question:

$$\mathrm{Pr}(B=1)=\Phi \left(\beta_{1}D+\beta_{2}S+\beta_{3}X+\beta_{4}M+\beta_{5}\mathrm{Ti}\right)$$

where Pr represents probability; *β*_*i*_ s are the parameters to estimate; *D* represents the sociodemographic characteristics of respondents; *S* represents health satisfaction of the respondent; *X* represents the properties of the health state using health state (H), duration (T), lead time in full health (L), person perspective (P), and satisfaction level (S) (see Figure 1 and Table 1); *M* represents the mode of administration; and Ti represents the time taken to complete the experimental question module. The function Φ(·) is the distribution function of the standard normal distribution [26,27]. Marginal effects are reported as they can be interpreted as percentages. For example, a marginal effect of−0.2 for a male indicates that being a male reduces the probability of choosing scenario B by 20%. For the regressions, statistical significance levels of both *P* less than 0.05 and *P* less than 0.01 are reported.

## Results

### Respondent Characteristics and Self-Reported Health Status

In total, 422 respondents completed either the online survey or the CAPI version. For the online survey, 2326 panel members were invited to take part and 487 potential respondents (20.1%) accessed the survey. Of these, 266 (11.4% of those invited; 54.6% of those accessing the survey) were screened out because they belonged to a completed age and sex quota (n = 158; 6.7% of those invited; 32.4% of those accessing), left the survey during completion (n = 19; 0.8% of those invited; 3.9% of those accessing), or completed the survey in less than 5 minutes (n = 108; 4.6% of those invited; 22.2% of those accessing). This group was defined as noncompleters. In total, 221 (9.5% of those invited; 46% of those accessing) fully completed the survey in 5 minutes or more. There were no significant age or sex differences between the responder and nonresponder samples.

The CAPI version was completed by 201 respondents. Information about the response rate for the survey is not available, because it was not recorded by the survey company, and therefore the response rate cannot be calculated. Nevertheless, in a similar CAPI study using the same methodology as part of the PRET project, Mulhern et al [28] found an overall response rate of 17.2%, and there is no reason to suggest that the response rate in this study would be substantially lower or higher than this. No CAPI respondents were excluded for completing the survey too quickly, and no respondents asked to stop the survey once they had begun answering the questions.

There were no significant differences between the online and CAPI groups by age and sex, but a number of demographic variables significantly differed between the samples (Table 2). These included employment status (with more retired people and homemakers in the CAPI sample, but more students in the online sample), marital status (with more CAPI respondents being married and more online respondents being single), and education level (with online respondents being educated to a higher level). In comparison to the general population, the overall, online, and CAPI samples all differ in terms of employment status, with more employed people in the general population (all *P*<0.001). The online sample is more similar to the general population in terms of marital status than the CAPI sample (with more of the CAPI sample being married or with a partner). The overall education level of the CAPI sample, however, is more similar to that of the general population.

In terms of the mean completion times, the CAPI sample took significantly longer to complete the overall survey, the background question, and self-reported health components of the survey, as well as experimental module 1 (five type I binary choice questions) and module 2 (one of the types II–VI binary choice questions). The difference between the samples for the time taken to complete module 3 (two DCE_TTO_ questions) was not significant. Across all three modules, the SD of the time taken is longer for the online sample than for CAPI sample. In terms of the median completion times, the CAPI sample took significantly longer to complete the overall survey, background questions, and all three experimental question modules (see Table 2).

**Fig. 2:**
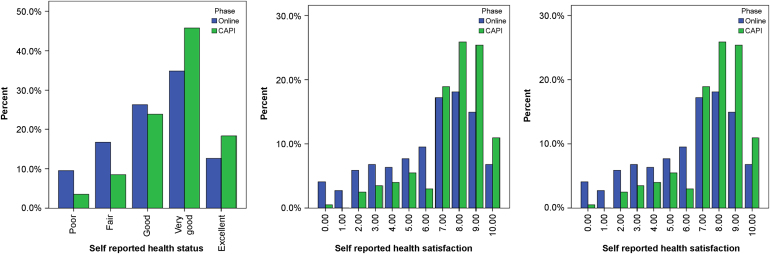
Self-reported health status and health and life satisfaction.

Responses to the self-report general health and health and life satisfaction questions are displayed in Figure 2. The CAPI sample are significantly more likely to report better health (*P*<0.001), higher levels of health satisfaction (*P*<0.001), and higher levels of life satisfaction (*P*<0.001). The mean EQ-5D-5L index score for the online sample (mapped from the EQ-5D-3L by using the algorithm produced by van Hout et al [29]) was 0.776 (0.25) and for the CAPI sample was 0.874 (0.20). This difference was significant (*F*(1, 409) = 18.66; *P*<0.001). EQ-5D-5L dimension responses also differ significantly by administration mode except mobility, with the CAPI group reporting less problems.

### Binary Choice Valuation Questions

The proportion of the sample choosing scenario B (i.e., choosing to live for a shorter duration in full health, choosing immediate death, or choosing the EQ-5D-5L state and associated duration for DCE_TTO_ questions) did not significantly differ by administration mode for any of the seven binary choice question types (Table 3). This was irrespective of the experimental attributes varied in the scenario. The result of equivalence across the scenarios is also consistent by using a range of different minimum time cutoff points for the overall survey (5, 6, 7, and 8 minutes) and also when dividing the sample into two groups on the basis of the time taken to complete each experimental module (group 1 included those completing the module in less than the median time taken to complete the module, and group 2 included those completing the module in more than or equal to the median time taken).

Probit regressions for each question reveal that a range of demographic and experimental attribute variables significantly predict the likelihood of choosing scenario B for a number of binary choice questions at the 0.05 level, but there is no pattern to the findings (Table 4). The mode of administration, the time taken to complete the question module, or the interaction between mode and completion time do not significantly predict responses across any of the question types.

For type I questions, response is significantly predicted by the health state and duration used in the question, where the more severe the health state or the larger the duration, the more likely scenario B is selected. These results cannot be tested across the other question types, as types II to VI include only one health state and associated duration. For type II questions, females are 5% and those with higher levels of life satisfaction are 5% more likely to choose to live in full health. For type IV, females are 11% and respondents who are retired are 13% more likely to choose scenario B. For type V, males are 12% and those who are not married or with a partner are 11% more likely to choose scenario B. Responses to some of the type VII questions are predicted by marital and employment status and life satisfaction, but these results are difficult to interpret owing to the nature of type VII questions, which presents two full EQ-5D-5L health states. Responses to question types III and VI are not predicted by any of the variables.

## Discussion

When health state valuation techniques such as TTO and SG were developed, face-to-face interviews were seen as the best way to administer the exercises, and this is the mode used to derive preferences for generic preference-based measures of health such as the EQ-5D questionnaire [1] and the six-dimensional health state short form (derived from SF-36) [2,3], as well as condition-specific instruments [30–32]. In recent years, there have been advances in communication technology and interest in the use of online health state valuation techniques is increasing. In parallel to this, health state valuation methods that are amenable to online administration, based on binary choice questions, have been developed [8,14,33]. There are issues, however, regarding the quality of data generated using online surveys. This article reports on a comparison between an identical set of binary choice questions designed to test issues related to health state valuation conducted in online and face-to-face environments. The results suggest that there is no difference between the responses to a range of binary choice valuation tasks across the administration modes. We also investigated the sample characteristics and found some differences between the groups. The finding of equivalence across the modes, however, remained robust after controlling for differences in the sample characteristics.

The responses to the main binary choice valuation questions were not statistically significantly different across the modes of administration, and this finding was not influenced by the severity or duration of the state used in the binary choice question. This is in comparison to a study comparing an iterative valuation technique (TTO) that found differences between online and face-to-face responses and concluded that this was due to the iterative nature of the process [6]. This is possibly because respondents who intend to complete the questions quickly may accept the first trade-off offered to avoid going through the process to reach indifference. Our study did not test an iterative process, but rather binary choice health state valuation tasks, which are amenable to online administration. The outcome has been that where a design that is suited to online and CAPI administration is used, comparable results are generated so that we cannot reject the hypothesis that the mode of administration does not affect the results. Furthermore, this result holds across different types of binary choice questions. The results, however, are based on a design where each type of question was asked only a handful of times. To infer from here that the outputs from online and CAPI versions of an actual valuation study where respondents are required to answer a larger number of questions of the same type will be no different, we would need to assume further that the effect of boredom caused by answering a larger number of similar questions is the same across the two modes of administration, which is something beyond the scope of this study. In other words, this study provides necessary but not sufficient evidence that the mode of administration may have little effect.

The finding of equivalence across modes of administration is valid for samples recruited following the standard procedures for CAPI (i.e., knocking on doors in selected postcode areas to produce a representative sample) and online (i.e., using participant panels) who were found to have similarities with the general population (the group targeted in most preference elicitation studies conducted in the world [1–5,8]). This demonstrates the potential applicability of our results in the design of valuation studies using binary methods. Nevertheless, it is unclear how these findings relate to other preference elicitation tasks. Equivalence at the binary level is necessary for equivalence at the iterative level. Nevertheless, our results are not sufficient to make any claims about equivalence at the iterative level. It may be possible to extend our findings to other valuation methods, and further work should consider the stability of a range of both iterative and binary choice preference elicitation techniques across different administration modes.

We have also assessed the time taken to complete the survey. If an online respondent completes the survey too quickly or too slowly, this may suggest that they are not fully engaged. The results demonstrate that the CAPI sample took significantly longer to complete the overall survey and two of the experimental modules. This may be because an interviewer is present and reads out the questions, and it is unlikely for the respondent to complete the survey without some minimal level of engagement. The shorter completion time data indicate that it is possible that at least some respondents in the online sample completed the survey without fully paying attention or engaging in the task. To try to counter this, we imposed a minimum time limit of 5 minutes for online respondents to be classified as completers. Five minutes was chosen from inspecting the completion times from the initial launch of the survey. The finding of equivalence across the modes of administration is consistent by using a range of cutoff points higher than 5 minutes and also by splitting the sample using the time taken to complete each module (which allows us to compare those completing each module quickly and those completing each module more slowly). This suggests that our findings are relatively robust. We have been unable, however, to assess the consistency of the results by using overall time cutoff points below 5 minutes, and this limits the wider applicability of the findings to binary choice valuation studies conducted online that do not impose minimum completion times. Future research may investigate respondent engagement in the online environment in more detail, for example, by analyzing responses using a wider range of minimum time limits, recording the time taken to complete each task, or developing innovative methods for presenting the tasks.

There have been concerns about the representativeness of online samples and how this might affect the comparability of results across samples [15,20,21]. The two samples in our study were recruited against age and sex quotas and therefore do not differ in terms of these characteristics. The two samples, however, differ significantly in some observable characteristics, and this raises the issue of representativeness with respect to the UK general population. Compared with previous census data [25], the CAPI samples are more representative in terms of educational attainment. The online sample overrepresents people educated to at least degree level, and this has also been found in other studies comparing online research groups with the general population [20]. Those who are educated to a higher level may be more computer literate, and this possibly explains their overrepresentation in the sample. In terms of health, it is possible that the online sample is genuinely less healthy than the CAPI sample. It has also been established, however, that individuals may answer face-to-face surveys in a socially desirable way, particularly when answering questions about sensitive issues such as mental health [34]. This may vary according to whether responses were public or anonymous [35]. In the CAPI sample, there may be a discrepancy between actual health and reported health status because of the presence of the interviewer, which may mean that, from the respondent’s perspective, responses are not completely anonymized. This did not, however, affect responses to the health state valuation questions.

We were not able to assess how the mode of administration affects the responses of those older than 65 years, because this group was not included in the sampling frame for the study. This potentially limits the applicability of our findings, as the Office of National Statistics predicts that approximately 59% of the adults 65 years or older use the Internet every day or almost every day (in comparison to ∼80% of those aged 18–54 years and 75% of those aged 55–64 years) [36]. Robinson et al. [10] included members of the population older than 65 years in their comparison of PTO tasks across different modes of administration and found that response to the Internet arm of the study was lower in this group. They did not, however, investigate differences in responses to the PTO task across different age groups. Further comparisons of valuation tasks across different modes of administration should investigate responses among those older than 65 years. This will establish the level of equivalence of health state valuation exercises across different modes of administration for the overall adult population.

It is in theory possible to make two samples agree in terms of any observable characteristic. Nevertheless, even with highly selective screening, the samples may differ in terms of further unobserved characteristics. The CAPI sample characteristics are influenced by who is at home when the interviewer visits, who agrees to take part, and who completes the interview. The online sample using an Internet panel is affected by who has access to the Internet, who is a member of the online panel, who in the panel agrees to take part, and who of those agreeing to take part completes the survey. It is not clear how the different selection mechanisms affect unobservable sample characteristics and therefore responses to health state valuation questions. Not everyone is equally likely to join an online panel and complete a particular survey or take part in a face-to-face interview. Typically, characteristics of nonresponders to interviews are not available, and one advantage of online surveys using existing Internet panels is that certain characteristics of nonresponders may be accessible. This allows for further insight into issues around nonresponse. A related issue is comparing the overall response rates for each mode of administration. Unfortunately, this study cannot inform this issue as the number of people who were invited to take part in the face-to-face CAPI study was not recorded by interviewers.

When designing health state valuation studies, the financial and time costs of the surveys are important and must be considered in light of the available preference elicitation methods and the modes available for administering the techniques. The cost of any survey has a fixed element and is not completely proportionate to the sample size. Nevertheless, even if generalized cost estimates cannot be given, it is generally the case that face-to-face interviews are substantially more costly per respondent and may take a much longer time to recruit sufficient numbers of participants. At the same time, any survey is only as good as the quality of the sample, and therefore the quality of the Internet panel needs to be scrutinized: one way to do this may be to look at how panel members are recruited and what incentives are offered. Overall, when the survey design is amenable to online administration, the incremental cost-effectiveness of conducting interview surveys must be examined.

This article discusses the findings from a head-to-head comparison of online and CAPI administrations of binary choice health state valuation questions. The two administrations have different advantages and disadvantages, and the two samples significantly differ across selected background characteristics, but the similarities with the general population indicate that the standard sampling frames used for face-to-face and online research studies are both valid. Responses to the main experimental binary choice questions, however, were not significantly different across the modes, and the mode of administration was not a significant factor explaining the responses. Therefore, both modes produce comparable data, and both can be used to administer health state valuation surveys including binary choice valuation questions such as DCE_TTO_ [8]. The advantages and disadvantages of both modes must be considered when designing health state valuation studies.

## Figures and Tables

**Table 1:** The 12 experimental questions used for the survey.

**Question type**	**Scenario A**					**Scenario B**	
	**H**	**T**	L	**P**	**S**	**H**	**T**
*Module 1*							
I	Slight problems walking about	10 y	n/m	You	n/m	Full health	9 y
I	Slight pain	10 wk	n/m	You	n/m	Full health	8 wk
I	Unable to walk about	10 y	n/m	You	n/m	Full health	8 y
I	Extreme pain	2 y	n/m	You	n/m	Full health	5 y
I	Extremely depressed	1 y	n/m	You	n/m	Full health	7 mo
*Module 2*							
II	Extreme pain	10 y	n/m	Somebody else	n/m	Full health	6 y
III	Slight pain	10 wk	10 wk	You	n/m	Full health	19 wk
IV	Extremely depressed	1 y	10 wk	Somebody else like you	n/m	Full health	7 mo
V	Unable to walk about	5 y	n/m	You	High	Full health	3 y
VI⁎	55555	10 y	10 yr	You	n/m	Immediate death	n/a
*Module 3*							
Subversion 1							
VIIa⁎	24144	5 y	n/m	You	n/m	54514	1 y
VIIb⁎	53543	10 y	n/m	You	n/m	31354	10 y
Subversion 2							
VIIc⁎	25555	1 y	n/m	You	n/m	42424	1 y
VIId⁎	41234	1 y	n/m	You	n/m	14112	1 y

n/m, not mentioned in scenario.

⁎ EQ-5D-5L health state listed.

**Table 2:** Sample characteristics.

**Characteristic**	**Overall**	**Online**	**CAPI**	**General population**	***P*****value**				
**Online, CAPI, and GP**	**Online vs. CAPI**	**Overall vs. GP**	**Online vs. GP**	**CAPI vs. GP**
n	422	221 (52.37)	201 (47.63)	Matched					

Age									
Mean±SD	41.49±13.96	41.56±14.38	41.41±13.52	42.23	n/a	0.913	n/a	n/a	n/a
Range	18–65	18–65	18–65	18–64					
Age category, n (%)					0.411	0.233	0.415	0.154	0.980
18–24	64 (15.2)	34 (15.4)	30 (15.0)	13.7					
25–34	97 (23.0)	51 (23.1)	46 (22.9)	23.2					
35–44	85 (20.1)	36 (16.29)	49 (24.4)	24.3					
45–54	86 (20.4)	46 (21.8)	40 (19.9)	21.6					
55–64	90 (21.3)	54 (24.4)	36 (17.9)	17.2					
Sex: Male, n (%)	201 (47.6)	102 (46.2)	99 (49.3)	47.9	0.836	0.524	0.945	0.703	0.765
Employment, n (%)					0.001	0.223	0.713	0.589	0.845
In employment	245 (58.1)	128 (57.9)	117 (58.2)	62					
Student	36 (8.5)	23 (10.4)	13 (6.5)	7					
Not in employment	141 (33.4)	70 (31.7)	68 (33.8)	31					
Marital status, n (%)					0.047	0.013	0.297	0.705	0.044
Married/partner	236 (55.9)	111 (50.7)	125 (62.2)	52.6					
Single	184 (43.6)	108 (49.3)	76 (37.8)	47.4					
Education cont after minimum age, n (%)	292 (69.2)	174 (78.7)	118 (58.7)	n/a	n/a	0.001	n/a	n/a	n/a
Educated to degree level, n (%)	136 (29.9)	90 (40.7)	46 (22.9)	21.6	0.001	0.032	0.001	0.001	0.719
Health status, n (%)					n/a	0.001	n/a	n/a	n/a
Good	340 (80.6)	163 (73.8)	177 (88.1)	n/a					
Poor	82 (19.4)	58 (26.2)	24 (12.0)	n/a					
Health satisfaction, n (%)					n/a	0.001	n/a	n/a	n/a
10	37 (8.8)	15 (6.8)	22 (11.0)	n/a					
6–9	279 (66.1)	132 (59.7)	147 (73.1)	n/a					
1–5	106 (25.1)	74 (33.5)	32 (15.9)	n/a					
Life satisfaction, n (%)					n/a	0.001	n/a	n/a	n/a
10	44 (10.4)	15 (6.8)	29 (14.4)	n/a					
6–9	266 (63.0)	131 (59.3)	135 (67.2)	n/a					
1–5	112 (26.5)	75 (33.9)	37 (18.4)	n/a					

Time taken to complete (min)									
Overall									
Mean±SD	9.88±4.6	8.64±3.84	11.26±4.99	n/a	n/a	0.001	n/a	n/a	n/a
Median	8.90	7.35	10.23	n/a	n/a	0.001	n/a	n/a	n/a

Nonexperimental questions									
Mean±SD	6.12±14.88	4.58±2.7	7.81±21.28	n/a	n/a	0.001	n/a	n/a	n/a
Median	4.48	3.80	5.10	n/a	n/a	0.001	n/a	n/a	n/a

Module 1									
Mean±SD	1.27±0.76	1.07±0.77	1.49±0.70	n/a	n/a	0.001	n/a	n/a	n/a
Median	1.05	0.87	1.35	n/a	n/a	0.001	n/a	n/a	n/a

Module 2									
Mean±SD	1.92±1.33	1.80±1.63	2.06±0.89	n/a	n/a	0.045	n/a	n/a	n/a
Median	1.68	1.42	1.87	n/a	n/a	0.001	n/a	n/a	n/a

Module 3									
Mean±SD	1.28±0.99	1.20±1.11	1.36±0.84	n/a	n/a	0.088	n/a	n/a	n/a
Median	1.05	0.92	1.22	n/a	n/a	0.001	n/a	n/a	n/a

*Note.* General population of England and Wales extracted for 18- to 65-year-olds from the 2001 census [25].

CAPI, computer-assisted personal interview; GP, general population.

**Table 3:** Proportion of respondents choosing scenario B in different binary choice questions by using 5-min cutoff and *P* values using other overall and within-module cutoffs.

**Question type**	**Proportions**		***P*****value**					
**5 min**			**6 min**	**7 min**	**8 min**
**Online (%)**	**CAPI (%)**	**Overall**	**Group 1**⁎	**Group 2**†			
**Sample size (overall n (online/CAPI)**	**221**	**201**	**422 (221/201)**			**351 (164/187)**	**298 (123/175)**	**246 (91/155)**

Module 1								
I	67.0	68.2	0.79	0.77	0.26	0.23	0.24	0.25
I	54.8	58.7	0.41	0.36	0.16	0.45	0.32	0.24
I	81.9	81.6	0.94	0.23	0.80	0.71	0.92	0.97
I	98.2	98.5	0.80	0.30	0.22	0.13	0.17	0.11
I	91.9	91.5	0.91	0.63	0.27	0.71	0.97	0.56

Module 2								
II	92.8	94.0	0.60	0.58	0.99	0.59	0.73	0.71
III	71.0	75.6	0.29	0.42	0.11	0.18	0.08	0.06
IV	81.9	83.1	0.75	0.61	0.81	0.67	0.73	0.62
V	56.6	60.7	0.39	0.09	0.68	0.60	0.72	0.75
VI	65.6	64.6	0.84	0.31	0.44	0.90	0.67	0.53

Module 3								
VIIa	41.4	39.0	0.77	0.20	0.08	0.78	0.72	0.55
VIIb	58.0	60.0	0.78	0.60	0.93	0.52	0.71	0.87
VIIc	77.6	71.0	0.37	0.94	0.09	0.74	0.51	0.31
VIId	78.0	82.2	0.54	0.52	0.83	0.27	0.39	0.24

*Note.* Types I–V, scenario B equals living in full health for a shorter duration; type VI, scenario B represents immediate death; type VII, scenario B is an EQ-5D-5L health state with duration.CAPI, computer-assisted personal interview.

⁎ Group 1: Those completing the module in less than the median time taken to complete the module. Sample size: module 1: 205 (149/56); module 2: 214 (139/69); module 3, type V1: 75 (38/37); module 3, type V2: 65 (25/40).

† Group 2: Those completing the module in more than or equal to the median time taken to complete the module. Sample size: module 1: 217 (72/145); module 2: 214 (82/132); module 3, type V1: 83 (20/63); module 3, type V2: 86 (25/61).

**Table 4:** Probit regression marginal effect coefficients for the likelihood of choosing scenario B.

**Variable**	**Type I**	**Type II**	**Type III**	**Type IV**	**Type V**	**Type VI**	**Type VIIa**	**Type VIIb**	**Type VIIc**	**Type VIId**
*Binary choice question components*										
Health state	0.09†	–	–	–	–	–	–	–	–	–
V value	0.05⁎	–	–	–	–	–	–	–	–	–
Duration of health state	0.03†	–	–	–	–	–	–	–	–	–

*Completion mode and time*										
Administration mode	0.02	0.00	0.11	0.01	0.20	0.08	0.12	0.18	0.10	0.06
Module completion time	−0.00	0.00	−0.00	−0.00	−0.00	0.00	0.00	0.00	−0.00	−0.00
Completion time×Mode interaction	0.00	0.00	−0.00	0.00	−0.00	−0.00	−0.00	−0.00	0.00	0.00

*Demographics*										
Sex	0.01	0.05⁎	−0.00	0.11⁎	−0.12⁎	0.04	0.06	−0.07	−0.10	0.05
Age	−0.01	0.00	−0.02	−0.00	−0.00	−0.00	−0.00	0.00	0.00	−0.00

Education level										
No education past minimum age	0.03	0.01	0.04	−0.08	−0.00	0.06	−0.05	0.02	−0.04	−0.09
Educated to degree level	−0.02	0.00	0.05	−0.04	−0.01	0.03	0.09	0.16	0.00	−0.06
Married or with partner	−0.03	0.00	−0.00	0.04	−0.11⁎	−0.02	−0.02	−0.02	0.24⁎	0.20⁎

Employment level										
Employed	−0.02	−0.02	0.02	−0.00	−0.05	0.06	0.07	−0.07	0.23⁎	0.10
Student	−0.05	0.01	0.01	0.5	−0.08	0.16	0.19	−0.00	0.35⁎	0.12
Not working	0.02	0.02	0.06	0.13⁎	−0.06	−0.04	−0.13	−0.12	0.24	0.16

Self-reported health										
Health status	0.00	0.01	−0.02	0.04	0.09	0.12	−0.24	0.07	0.06	0.01
Health satisfaction	−0.04⁎	0.02	−0.01	0.02	0.06	0.05	−0.09	−0.08	−0.12	0.01
Life satisfaction	0.01	0.05⁎	−0.01	−0.07	0.01	−0.02	0.07	0.10	−0.21⁎	0.07

N	2105	422	422	422	422	422	157	157	149	149
LR χ^2^	369.94	9.56	10.62	21.93	17.73	15.64	12.08	15.49	16.19	11.81
Pseudo *R*^2^	0.17	0.05	0.02	0.06	0.03	0.03	0.05	0.09	0.08	0.08
Log likelihood	−888.84	−98.23	−239.88	−184.96	−277.47	−264.97	−99.70	−82.41	−92.73	−67.53

*Note.* Health state, V value, and duration can be analyzed only for type I questions.

⁎ Significant at *P* = 0.05.

† Significant at *P* = 0.01.

## References

[1] Dolan P. (1997). Modelling valuations for EuroQol health states. Med Care.

[2] Brazier J., Roberts J., Deverill M. (2002). The estimation of a preference-based measure of health from the SF-36. J Health Econ.

[3] Brazier J.E., Roberts J. (2004). Estimating a preference-based index from the SF-12. Med Care.

[4] Wittrup-Jensen K.U., Lauridsen J., Gudex C., Pedersen K.M. (2009). Generation of a Danish TTO value set for EQ-5D health states. Scand J Public Health.

[5] Viney R., Norman R., King M.T. (2011). Time trade off EQ-5D weights for Australia. Value Health.

[6] Norman R., King M.T., Clarke D. (2010). Does mode of administration matter? Comparison of online and face-to-face administration of a time trade-off task. Qual Life Res.

[7] Brazier J., Rowen D., Yang Y., Tsuchiya A. (2012). Comparison of health state utility values derived using time trade-off, rank and discrete choice data anchored on the full health-dead scale. Eur J Health Econ.

[8] Bansback N., Brazier J., Tsuchiya A., Anis A. (2012). Using a discrete choice experiment to estimate societal health state utility values. J Health Econ.

[9] Dolan P., Gudex C., Kind P., Williams A. (1996). Valuing health states: a comparison of methods. J Health Econ.

[10] Robinson A., Covey J., Jones-Lee M., Loomes G. (2008). Comparing the Results of Face-to-Face and Web Based PTO Exercises.

[11] Damschroder L.J., Baron J., Hershey J.C. (2004). The validity of person tradeoff measurements: randomized trial of computer elicitation versus face-to-face interview. Med Decis Making.

[12] Rowen D., Brazier J., van Hout B. (2011). A comparison of methods for converting DCE values onto the full health-dead QALY scale. HEDS Discussion Paper, 11/15.

[13] van Hout B., Oppe M. (2011). Combining DCE and TTO into a single value function. Value Health.

[14] Bosch J.L., Kammitt J.K., Weinstein M.C., Hunink M.G.M. (1998). Estimating general-population utilities using one binary-gamble question per respondent. Med Decis Making.

[15] Schmidt W.C. (1997). World Wide Web survey research: benefits, potential problems, and solutions. Behav Res Methods Instrum Comput.

[16] Eysenbach G. (2005). The law of attrition. J Med Internet Res.

[17] Bowling A. (2005). Mode of questionnaire administration can have serious effects on data quality. J Pub Health.

[18] Rieps U., Birnbaum M.H. (2000). The web experiment method: advantages, disadvantages, and solutions. Psychological Experiments on the Internet.

[19] Strickland O.L., Moloney M.F., Dietrich A.S. (2003). Measurement issues related to data collection on the World Wide Web. ANS Adv Nurs Sci.

[20] Liu H., Cella D., Gershon R. (2010). Representativeness of the patient-reported outcomes measurement information system internet panel. J Clin Epidemiol.

[21] Schillewaert N., Meulemeester P. (2005). Comparing response distributions of offline and online data collection methods. Int J Market Res.

[22] Raat H., Mangunkusumo R.T., Landgraf J.M. (2007). Feasibility, reliability, and validity of adolescent health status measurement by the Child Health Questionnaire Child Form (CHQ-CF): Internet administration compared with the standard paper version. Qual Life Res.

[23] Tsuchiya A., Mulhern B. (2011). Preparation for the Re-valuation of the EQ-5D Tariff (PRET) project: overview of methods for project stages 1-3. HEDS Discussion Paper.

[24] Herdman M., Gudex C., Lloyd A. (2011). Development and preliminary testing of the new five-level version of EQ-5D (EQ-5D-5L). Qual Life Res.

[25] Office of National Statistics. Census 2001: General Report for England and Wales. South Wales, UK: Office of National Statistics, 2005.

[26] Alexandre P.K., French M.T. (2004). Further evidence on the labor market effects of addiction: chronic drug use and employment in metropolitan Miami. Contemp Econ Policy.

[27] Greene W.H. (2000). Econometric Analysis.

[28] Mulhern B., Tsuchiya A., Devlin N. (2012). A Comparison of Three Binary Choice Methods for Health State Valuation.

[29] van Hout B., Janssen M.F., Feng Y.S. (2012). Interim scoring for the EQ-5D-5L: mapping the EQ-5D-5L to EQ-5D-3L value sets. Value Health.

[30] Rowen D., Mulhern B., Banerjee S. (2012). Estimating preference based single index measures for dementia using DEMQOL and DEMQOL-Proxy. Value Health.

[31] Yang Y, Brazier J, Tsuchiya A, Coyne K., Estimating a preference-based index from the Overactive Bladder questionnaire. Value Health 2008;12:59–66. 10.1111/j.1524-4733.2008.00413.x 18647258

[32] Yang Y., Brazier J.E., Tsuchiya A., Young T. (2011). Estimating a preference based index for a 5-dimensional health state classification for asthma derived from the asthma quality of life questionnaire. Med Decis Making.

[33] Ratcliffe J., Couzner L., Flynn T. (2011). Valuing Child Health Utility 9D health states with a young adolescent sample: a feasibility study to compare Best-Worst Discrete Choice Experiment, Standard Gamble and Time Trade Off methods. Appl Health Econ Health Policy.

[34] Robling M.R., Ingledew D.K., Greene G. (2010). Applying an extended theoretical framework for data collection mode to health services research. BMC Health Serv Res.

[35] Paulhus D.L. (1984). Two-component models of socially desirable responding. J Pers Soc Psychol.

[36] Office of National Statistics. Internet Access 2010: Households and Individuals. South Wales, UK: Office of National Statistics, 2010.

